# Integrated Approach for Testing and Assessment for Developmental Neurotoxicity (DNT) to Prioritize Aromatic Organophosphorus Flame Retardants

**DOI:** 10.3390/toxics12060437

**Published:** 2024-06-18

**Authors:** Anna Kreutz, Oluwakemi B. Oyetade, Xiaoqing Chang, Jui-Hua Hsieh, Mamta Behl, David G. Allen, Nicole C. Kleinstreuer, Helena T. Hogberg

**Affiliations:** 1 Inotiv, Research Triangle Park, NC 27560, USA; anna.kreutz@inotivco.com (A.K.); oluwakemi.oyetade@inotivco.com (O.B.O.); xiaoqing.chang@inotivco.com (X.C.); dallen@iccs-cosmetics.org (D.G.A.); 2 NIH/NIEHS/DTT/PTB, Research Triangle Park, NC 27560, USA; jui-hua.hsieh@nih.gov; 3 Neurocrine Biosciences Inc., San Diego, CA 92130, USA; mbehl@neurocrine.com; 4 NIH/NIEHS/DTT/NICEATM, Research Triangle Park, NC 27560, USA; nicole.kleinstreuer@nih.gov

**Keywords:** developmental neurotoxicity, integrated approach to testing and assessment, organophosphorus flame retardants keyword, new approach methodologies, in vitro battery, physiologically-based toxicokinetic models

## Abstract

Organophosphorus flame retardants (OPFRs) are abundant and persistent in the environment but have limited toxicity information. Their similarity in structure to organophosphate pesticides presents great concern for developmental neurotoxicity (DNT). However, current in vivo testing is not suitable to provide DNT information on the amount of OPFRs that lack data. Over the past decade, an in vitro battery was developed to enhance DNT assessment, consisting of assays that evaluate cellular processes in neurodevelopment and function. In this study, behavioral data of small model organisms were also included. To assess if these assays provide sufficient mechanistic coverage to prioritize chemicals for further testing and/or identify hazards, an integrated approach to testing and assessment (IATA) was developed with additional information from the Integrated Chemical Environment (ICE) and the literature. Human biomonitoring and exposure data were identified and physiologically-based toxicokinetic models were applied to relate in vitro toxicity data to human exposure based on maximum plasma concentration. Eight OPFRs were evaluated, including aromatic OPFRs (triphenyl phosphate (TPHP), isopropylated phenyl phosphate (IPP), 2-ethylhexyl diphenyl phosphate (EHDP), tricresyl phosphate (TMPP), isodecyl diphenyl phosphate (IDDP), tert-butylphenyl diphenyl phosphate (BPDP)) and halogenated FRs ((Tris(1,3-dichloro-2-propyl) phosphate (TDCIPP), tris(2-chloroethyl) phosphate (TCEP)). Two representative brominated flame retardants (BFRs) (2,2′4,4′-tetrabromodiphenyl ether (BDE-47) and 3,3′,5,5′-tetrabromobisphenol A (TBBPA)) with known DNT potential were selected for toxicity benchmarking. Data from the DNT battery indicate that the aromatic OPFRs have activity at similar concentrations as the BFRs and should therefore be evaluated further. However, these assays provide limited information on the mechanism of the compounds. By integrating information from ICE and the literature, endocrine disruption was identified as a potential mechanism. This IATA case study indicates that human exposure to some OPFRs could lead to a plasma concentration similar to those exerting in vitro activities, indicating potential concern for human health.

## 1. Introduction

Brominated flame retardants (BFRs) are historically the most commonly used group of flame retardants, which contain a mixture of diverse chemicals. They are produced in large quantities and popularly used to reduce the flammability of combustible materials and in many other industrial processes [1]. Owing to these applications, BFRs have become ubiquitous in the environment, but due to concerns of their toxicity and bioaccumulation potency, they have been phased out, leading to replacement with alternative chemicals like the organophosphorus flame retardants (OPFRs). OPFRs have since then become persistent in the environment and are now found at even higher exposure levels due to extensive usage [2]. OPFRs are additive components, so do not chemically bind to products and can therefore be released from the polymer matrix through leaching, evaporation, migration, and abrasion. Exposure of humans to OPFRs can occur through inhalation of air, dermal contact, ingestion of dust, or intake of contaminated food or drinking water [3]. Some scientific evidence from in vivo and in vitro studies suggests that OPFRs may be hazardous, and that exposure may lead to adverse health effects. However, there is not enough toxicity information to provide a regulatory hazard assessment of OPFRs.

In 2019, a report from the National Academy of Science (NAS), emphasized the need for a class evaluation of flame retardants in traditional animal guideline studies, as there are far too many to be tested one by one [4]. The report also recognized that some of the main issues with the traditional approach are that chemicals lacking adequate toxicological data are typically treated as non-hazardous, while cumulative exposure and risk are largely ignored. In order to make more informed decisions regarding chemical substitutions and better regulate the tens of thousands of chemicals found in commerce, higher-throughput strategies are needed [5,6].

**Figure 1:**
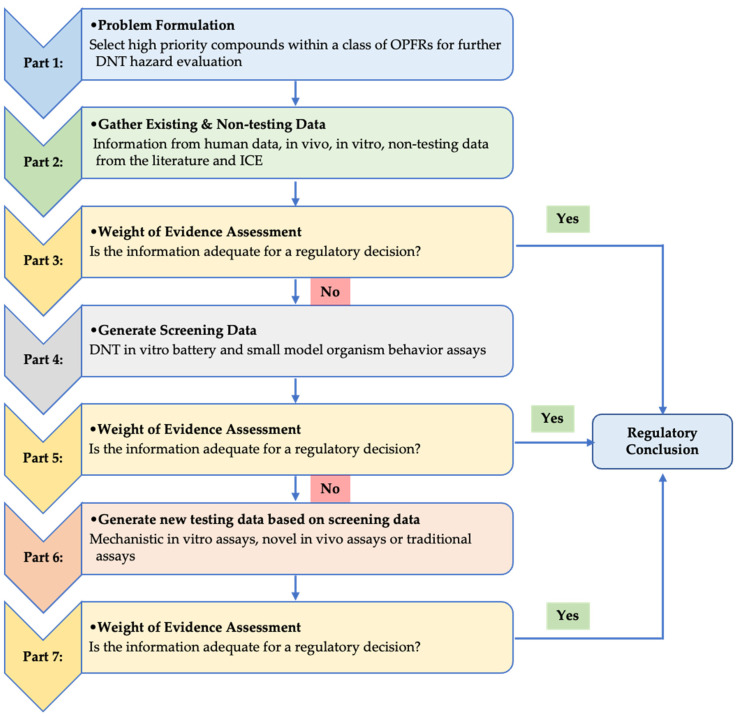
Integrated Approaches to Testing and Assessment (IATA) flowchart for prioritization of chemicals for further DNT testing. (1) The first step in the IATA is to define problem the formulation that, in this case, was to select high priority compounds within the class of aromatic organophosphorus flame retardants (OPFRs) for further DNT hazard evaluation. (2) Secondly all available data were gathered, and (3) a weight of evidence (WoE) assessment was performed. Due to an inefficient amount of information for a regulatory decision (4), screening level information was generated using a battery of in vitro assays and zebrafish embryo behavior assays. (5) A new WoE assessment was performed to select high priority compounds within the aromatic OPFRs that then (6) should be tested further for DNT effects using either more mechanistic in vitro or in vivo assays. More testing data should be generated until (7) the WoE assessment concludes that adequate information has been provided to make a regulatory decision.

A petition to the U.S. Consumer Product Safety Commission (CPSC) to ban several flame-retardant products was submitted by a coalition of organizations and individuals to initiate a regulatory action under the U.S. Federal Hazardous Substances Act (FHSA). To determine if a ban would be enacted, the U.S. CPSC must firstly perform a hazard assessment to evaluate whether the chemical is toxic, as defined by the U.S. FHSA. Accordingly, OPFRs were nominated by the U.S. CPSC to the National Toxicology Program (NTP), National Institute of Environmental Health Sciences (NIEHS), for testing based on predicted increased exposure and lack of hazard characterization data. OPFRs are structurally similar to organophosphorus insecticides that are well-established (developmental) neurotoxicants [7,8]. Combined with the developing brain being especially vulnerable to chemical perturbation, this makes this compound class of high concern for developmental neurotoxicity (DNT) effects [9]. The traditional DNT in vivo guideline study takes years to conduct, is expensive, and requires the use of large numbers of animals—an untenable approach for screening large numbers of compounds. Further, the animal studies are not always reflective of the developing nervous system in toddlers and children who are at an increased risk of exposure to these chemicals due to hand-to-mouth behavior [10,11]. Over the past decade, great efforts from the regulatory, scientific, and stakeholder communities have been aimed at developing a DNT in vitro battery (IVB) to enhance DNT assessment [12,13,14]. This battery captures a variety of processes critical to neurodevelopment in vivo, such as cell proliferation, migration, differentiation, synaptogenesis, and network formation and function. The eventual goal of this effort has been to offer a suite of assays that could inform on DNT hazards for regulatory agencies. Recently, the OECD guidance document “Initial Recommendations on Evaluation of Data from the Developmental Neurotoxicity (DNT) In Vitro Testing Battery” [15] was published with specific Integrated Approaches to Testing and Assessment (IATA) case studies demonstrating the application and interpretation of the DNT testing battery [16]. The IATA framework was developed by the OECD and allows integration of all available data to be used in regulatory assessment [17]. Hence, an IATA case study was developed for OPFRs to demonstrate how a DNT–IVB combined with behavior assays in small model organisms (here after referred as the DNT battery) can be applied to prioritize a class of chemicals for further testing (Figure 1).

In addition to these assays, the scientific literature and the Integrated Chemical Environment (ICE) [18] database were evaluated for data on endpoints and processes not covered in the DNT battery, such as glia differentiation and function, ontogeny of neurotransmitters and receptors, and endocrine disruption. Exposure information was extracted from the literature and compared to the in vitro data using physiologically-based pharmacokinetic (PBK) models to assess potential human risks.

## 2. Materials and Methods

### 2.1. Chemicals

Six aromatic OPFRs with limited DNT information were prioritized for further testing (Table 1) based on nomination to the NTP by the U.S. CPSC on account of anticipated exposure increases and the need for hazard characterization data. Representative BFRs and halogenated OPFRs were selected to compare relative toxicity (Table 1). All chemicals were supplied by the Division of Translational Toxicology (DTT), NIEHS. Stock solutions were prepared in DMSO and tested in a broad range of concentrations (0.01 to 20 µM, up to 100 µM for planaria) for most assays. Further details on chemical sourcing and purity are provided in Supplemental Table S1. Chemical exposures were generally applied in a final DMSO concentration of 0.1%, apart from 0.46% DMSO for NCATS neurite outgrowth assays, and the behavioral assays, which were performed using a final DMSO concentration of 0.5–0.64% DMSO.

### 2.2. Assays in the DNT Battery

Twenty-two new approach methodologies (NAMs) (eighteen in vitro and four small model organism behavior assays) that measure DNT or acute neurotoxicity (NT) critical processes were included in this case study. The assays and relevance of the processes for DNT are described below. Further details can be found in the respective publications for assays (Table 2).

#### 2.2.1. Proliferation

Proliferation of neural progenitor cells (NPCs) is an important key process that if perturbed, can alter the number of cells in the CNS and lead to neurodevelopmental disorders/disturbances, e.g., microcephaly [19,20]. Two assays were applied to measure proliferation.

i. Proliferation@IUF

Human NPCs (hNPCs) of fetal origin (Lonza) from three different individuals (gestational week 16–19) were cultured as free floating neurospheres in proliferation medium [21]. Proliferation of primary hNPCs grown as neurospheres was analyzed after 3 days of chemical exposure. Proliferation was assessed by bromodeoxyuridine (BrdU) incorporation, which was added 18 h prior to the end of the experiment. Following dissociation of the spheres, proliferation was assessed using a luminescence-based ELISA BrdU assay [22]. Cytotoxicity was assessed using the alamar blue viability assay.

ii. Proliferation@USEPA

Cell proliferation was determined in embryonic stem cell-derived hNP1 human neural progenitor cells by immunocytochemical assessment of BrdU incorporation into replicating DNA [23]. Briefly, cells were exposed to chemicals for 20 h before BrdU was added to the media for another 4 hours. Cells were then fixed and immunostained with a BrdU antibody. BrdU-positive cells were counted using high-content imaging on a Cellomics Arrayscan VTI (software: Target Activation Bioapplication V4) and divided by the number of total cells (stained with Hoechst 33258) to calculate the % of proliferating cells.

#### 2.2.2. Migration

Following proliferation, cells will migrate away from their site of origin near the ventricle to their final position in the brain. The cell migration process occurs at different time points for different cell types. As the migration process can continue for several months after birth, this critical process is sensitive to perturbations throughout an extended time period [24]. Migration was assessed for four different cell types, neural crest cells (NCCs), radial glia, neurons, and oligodendrocytes.

i. NCC migration@UKonstanz

Human embryonic stem cells (H9 line) were differentiated into neural rosettes containing NCCs, a cell population that appears during early neurodevelopment [25]. NCCs were sorted and seeded in wells containing silicone stoppers (Platypus Technologies) to create a cell free area. One day after seeding the stoppers were removed and the cells could migrate into the cell free area. After 24 h of migration, the cells were exposed to chemicals for an additional 24 h before the migration was assessed by imaging on a high content microscope (Cellomics ArrayScanVTI, Thermo Fischer, Waltham, Massachusetts, United States). Quantification of the migration was performed using a freely available software tool (http://invitrotox.unikonstanz.de/) (access date 15 April 2024).

ii. Radial glia migration@IUF

Initial rounds of proliferation were performed to increase the pool of primary neural progenitor cells (hNPCs) of fetal origin (Lonza). Cells were then cultured as neurospheres in suspension and transferred onto poly-D-lysine-laminin-coated 96-well plates to attach and initiate migration. During migration the NPCs differentiate into radial glia, neurons, and oligodendrocytes [21]. Radial glial migration distance was quantified manually based on the migration distance in brightfield images after 72 h. After 5 days of migration and differentiation during chemical exposure, human neurospheres were fixed and stained for various cell specific markers. Radial glia cells were identified based on immunocytochemical staining (ICC) for Hoechst-positive nuclei (furthest migrated cells/extensions are always radial glia, based on historical data). The migration area was assessed automatically using two convolutional neural networks (CNNs) based on the Keras architecture implemented in Python 3 and the Omnisphero software for image analysis [26]. Cytotoxicity was assessed using the alamar blue viability assay.

iii. Neuronal migration@IUF

Neuronal migration was assessed using the same neurosphere and plating protocol as for the radial glia migration@IUF assay [21]. Neuronal migration was quantified as the mean % distance of all βIII tubulin+ cells divided by the migration distance of radial glia at 120 h. Cytotoxicity was assessed using the alamar blue viability assay.

iv. Oligo migration@IUF

Oligodendrocyte migration was assessed using the same neurosphere and plating protocol as for the radial glia migration@IUF assay [21]. Oligodendrocyte migration was quantified as the mean % distance of O4+ cells divided by the migration distance of radial glia at 120 h. Cytotoxicity was assessed using the alamar blue viability assay.

#### 2.2.3. Differentiation

After the last cell division during the proliferation phase and during migration, NPCs start to differentiate into various cell types. At first, they give rise to different kind of neurons, distinct by e.g., their shape, size, polarity, and expression of neurotransmitters and receptors. At later neurogenic stages, NPCs also differentiate into astrocytes and oligodendrocytes [24]. Oligodendrocytes provide the critical function of myelinating axons in the central nervous system (CNS). As oligodendrocytes are operating near their metabolic capacity, they are particularly sensitive to chemical disturbances that may affect their differentiation and function [27]. Here, two assays were used to assess differentiation into neurons and oligodendrocytes.

i. Neuronal differentiation@IUF

Neuronal differentiation was assessed using the same neurosphere and plating protocol as for the radial glia migration@IUF assay [21]. NPCs differentiated into neurons after 120 h were assessed by automatically counting the number of βIII tubulin-positive cells within the migration area divided by the number of total nuclei (Hoechst 33258) using the CNN based on the Keras architecture implemented in Python 3 and the Omnisphero software for image analysis [26]. Cytotoxicity was assessed using the alamar blue viability assay.

ii. Oligodendrocyte differentiation@IUF

Oligodendrocyte differentiation was assessed using the same neurosphere and plating protocol as for the radial glia migration@IUF assay [21]. The number of O4-positive cells within the migration area was counted by using the Omnisphero platform [26] and divided by the number of total nuclei (Hoechst 33258) to calculate the % of differentiated oligodendrocytes. Cytotoxicity was assessed using the alamar blue viability assay.

#### 2.2.4. Neurite Outgrowth

During neuronal differentiation and maturation, axons and dendrites form a neuronal network, initially by elongation and branching of the neurites (outgrowth). Disturbance to this process can lead to impaired synaptogenesis and neuronal function. The complexity of network formation makes it a vulnerable process for chemical perturbation and any interference, e.g. with gene expression, membrane receptors and ion channels, or intracellular signaling, can affect the neurite elongation and growth [28]. Eight different assays (rodent, human, monolayer, neurospheres, peripheral nervous system (PNS), and CNS neurons) were applied to assess the neurite outgrowth.

i. Neurite outgrowth@USEPA (rat)

Rat primary mixed cortical cultures were prepared from postnatal day 0 Long-Evans rat pups and seeded at low density [23]. Cells were exposed 2 h postseeding to chemicals for 48 h, then fixed and stained for the neuronal marker βIII tubulin. Neurite outgrowth was assessed by measuring total neurite length per neuron, neurite count, and branch point count using high-content imaging on a Cellomics Arrayscan VTI (software: NeuronalProliferation Bioapplication, V4, 6.6.0). Viability was assessed by alamar blue and lactate dehydrogenase (LDH).

ii. Neurite outgrowth@MolDevices

Human iPSC-derived neurons (iCell), consisting of a mixture of postmitotic GABAergic and glutamatergic neurons, were provided by Cellular Dynamics International [29]. Cells were seeded at low density for 48 h prior to chemical treatment. After a 72 h exposure, the cells were stained with Calcein AM and Hoechst 33342. Images of the cells were acquired with the ImageXpress Micro XLS system (Molecular Devices, LLC, San Jose, CA, USA) and analyzed with MetaXpress5software (MolecularDevices, LLC, San Jose, CA, USA). Neurite outgrowth was characterized by the extent of outgrowth (e.g., length of total outgrowth and mean outgrowth per cell), the number of neurite processes (e.g., total number of processes and mean number of processes per cell), and the extent of branching (e.g., total number of branches and mean number of branches per cell).

iii. Neurite outgrowth@USEPA (human)

Human embryonic stem cell-derived neurons (hN2TM) obtained from ArunA Biomedical were seeded at low density and exposed to chemicals 2 h postseeding [23]. Assessment of the neurites was performed as the Neurite outgrowth@USEPA (rat) assay after 48 h exposure. Viability was assessed by alamar blue and lactate dehydrogenase (LDH).

iv. CNS neurite outgrowth@UKonstanz

Lund human mesencephalic cells (LUHMES) were differentiated into dopaminergic like neurons for 48 h [30]. Cells were then replated at a low density and differentiation was continued for 24 h in the presence of chemicals. Cells were stained with Calcein AM and Hoechst 33342 before being imaged on a Cellomics high-content imaging ArrayScan VTI microscope. To quantify neurite area, an automated algorithm was used, which calculates the area of the cell soma and subtracts this area from all calcein-positive pixels imaged.

v. PNS neurite outgrowth@UKonstanz

Human sensory neurons derived from hECSs (WA09 Line) were obtained from WiCell [30]. Cells were seeded at low density and exposed to chemicals for 24 h. Assessment of the neurites was performed as the CNS neurite outgrowth@UKonstanz assay.

vi. Neurite outgrowth@IUF

Neurite outgrowth@IUF was assessed using the same neurosphere, plating, and exposure protocol as for the radial glia migration@IUF assay [21]. Neurite length (in μm) and area (number of pixels) were calculated automatically for βIII tubulin-stained processes by the high-content image analysis (HCA) tool Omnisphero [26] after a 120 h exposure. Cytotoxicity was assessed using the alamar blue viability assay.

vii./iix. CNS and PNS neurite outgrowth@NCATS

iPSC-derived human spinal motor and human cortical glutamatergic neurons purchased from and labeled with GFP by BrainXell [31] were differentiated into motor or cortical neurons [32,33]. GFP-expressing human neurons were seeded at low density onto poly-D-lysine-coated 1536-well plates and incubated at 37 °C for 40 h to allow for cell attachment and growth prior to chemical exposure. Endpoints were assessed at 24 and 48 h after chemical exposure. Viability was assessed based on cell counts. Cytotoxicity was assessed using Calcein AM. Imaging was performed on an Operetta high-content imaging system (Perkin Elmer, Waltham, MA, USA). Neurite outgrowth was assessed based on total neurite length, number of segments, and maximum neurite length. Cell counts were also quantified as a measure of viability [34].

#### 2.2.5. Network Formation and Function

To achieve mature neuronal function, neurons must form cell–cell contacts through synapses. In spontaneously electrically active in vitro models, electrophysiological techniques can be used to evaluate functional synapses in developing and mature neuronal networks. Any disturbance to key processes prior and during neuronal network formation e.g., proliferation, migration, and differentiation, would likely also alter the electrical activity [13]. Two assays were used to assess network formation and neuronal function. Activity was recorded using an Axion Biosystems Maestro 768 channel amplifier and Axion Integrated Studios (AxIS) v1.7 software.

i. Acute neuronal firing@USEPA

Rat primary mixed cortical cultures were prepared from postnatal day 0 Long-Evans rat pups and plated into the center of each well of a 48 well micro-electrode array (MEA) plate [23]. On DIV 13 or 14, spontaneous network spiking activity (extracellularly recorded action potential “spikes”) was recorded. Activity was recorded prior to chemical exposure for 1 h (baseline), as well as for 1 h following chemical exposure. Chemical effects were expressed as a percentage of the baseline activity in each well following exposure. Viability was assessed by alamar blue and lactate dehydrogenase (LDH).

ii. Network formation@USEPA

Rat primary mixed cortical cultures were prepared from postnatal day 0 Long-Evans rat pups and plated into the center of each well of a 48 well MEA plate [35]. Cells were exposed to chemicals 2 h after seeding for 12 days. The spontaneous electrical activity was recorded for 15 min on DIV 2, 5, 7, 9, and 12. Various parameters were analyzed and potency values for chemicals that impacted network activity, including both decreased terminal DIV 12 activity and delayed development of activity over time. The temporal component of each well’s recordings was reduced to a single area under the curve (AUC) value [36]. Viability was assessed by alamar blue and lactate dehydrogenase (LDH).

#### 2.2.6. Behavior

Behavioral tests (e.g., locomotor activity) are considered critical for DNT risk assessment and are included in in vivo guideline studies, but are difficult to measure in vitro. Zebrafish at early developmental stages (0–5 days postfertilization (dpf), considered non-animal testing according to EU legislation) have shown their potential as a whole organism approach to predict human DNT and are included in this IATA. The fundamental principles of key cellular events during brain development are remarkably conserved across species [37,38], and the use of alternative species can allow for direct testing of cause–effect relationships between toxicant exposure and cellular and behavioral endpoints [39,40]. Similar locomotor assays using zebrafish were evaluated from three different laboratories. Planaria provide comparable advantages to zebrafish as they are small, develop quickly, and display a wide range of behaviors and are thus also included in this DNT screening approach.

i. Behavior@Biobide

Adult zebrafish from a wildtype AB strain were housed and maintained in accordance with a standard 14 h light/10 h dark cycle [41]. Embryos were exposed to chemicals at 3 dpf for 48 h, followed by placement in a DanioVision automated tracking system powered by EthoVision. Several parameters were analyzed including velocity, movement duration, and frequency of activity, but the total distance moved was selected as representative of locomotor activity. The mean total distance moved by larvae in each group was measured in 2 min time bins in two rounds of 10 min light and 10 min dark phases. Viability was assessed at the respective days and tested as percent affected.

ii. Behavior@OregonStateU

Tropical 5D wildtype adult zebrafish were housed at Oregon State University, Sinnhuber Aquatic Research Laboratory (SARL) in a standard 14 h light/10 h dark cycle [42]. Embryos were collected and cleaned at 4 h postfertilization (hpf), before chorions were enzymatically removed at 6 hpf and exposed to chemicals, followed by behavioral assessments at 5 dpf using Viewpoint LifeScience Zebraboxes [43,44]. The testing paradigm of the photomotor response assay consisted of four light cycles, each cycle consisting of 3 min of alternating light and dark. Viability was assessed at the respective days and tested as percent affected.

iii. Behavior@UCDavis

Tropical 5D wildtype adult zebrafish were obtained from SARL at Oregon State University (Corvallis, OR, USA) and subsequent generations were raised at UC Davis. Adult zebrafish were maintained under standard laboratory conditions in 14 h light/10 h dark cycle [45]. Embryos were collected and chorions were enzymatically removed and exposed to chemicals at 6 hpf. Photomotor behavior was assessed at 4 and 5 dpf using the DanioVision system. The testing paradigm consisted of four light cycles, with each cycle consisting of 5 min of alternating light and dark (last dark cycle 15 min). Larval movement was recorded using a GigE camera (Noldus) with an infrared filter and tracked using EthoVisionXT software (Noldus). The total distance swam in mm was measured in 1 min bins. Viability was assessed at the respective days and tested as percent affected.

iv. Behavior@UCSanDiego

Adult asexual D. japonica, originally obtained from China over 5 years ago, were cultivated in the Collins lab at UCSD [45]. Planarians were exposed 24 h after head amputation for a period of 12 days, with screening performed on days 7 and 12. Planaria were screened for mortality at days 7 and 12, eye regeneration at day 7, and behavior at days 7 (unstimulated and phototaxis) and 12 (unstimulated, phototaxis, scrunching, and thermotaxis). Viability was assessed at the respective days and tested for mortality.

**Table 2:** Assays represented in the DNT Battery.

Assay	Model	References
**Proliferation**		
Proliferation@IUF	Human 3D neurosphere	[21]
Proliferation@USEPA	Human hNP1	[23]
**Migration**		
NCC migration@UKonstanz	Human crest cells	[25]
Radial glia migration@IUF ^1^	Human 3D neurosphere	[21]
Neuronal migration@IUF ^1^	Human 3D neurosphere	[21]
Oligo migration@IUF ^1^	Human 3D neurosphere	[21]
**Differentiation**		
Neuron differentiation@IUF ^1^	Human 3D neurosphere	[21]
Oligodendrocyte differentiation@IUF ^1^	Human 3D neurosphere	[21]
**Neurite outgrowth**		
Neurite outgrowth@USEPA	Rat primary cortical	[23]
Neurite outgrowth@MolDevices	Human iPSC-derived	[29]
Neurite outgrowth@USEPA	Human hN2	[23]
CNS neurite outgrowth@UKonstanz	Human LUHMES	[30]
PNS neurite outgrowth@UKonstanz	Human ESC-derived	[30]
Neurite outgrowth@IUF ^1^	Human 3D neurosphere	[21]
CNS neurite outgrowth@NCATS	Human GFP-labeled cortical iPSCs	[34]
PNS neurite outgrowth@NCATS	Human GFP-labeled spinal iPSCs	[34]
**Firing/Network formation**		
Acute neuronal firing@USEPA	Rat primary cortical	[23]
Network formation@USEPA	Rat primary cortical	[35]
**Behavior**		
Behavior@Biobide	Zebrafish	[41]
Behavior@OregoneStateU	Zebrafish	[42]
Behavior@UCSanDiego	Planarian	[45]
Behavior@UCDavis	Zebrafish	[45]

^1^ Performed together.

### 2.3. Data Analysis

#### 2.3.1. Benchmark Concentration (BMC) Approach

For this case study, the benchmark concentration (BMC) approach was used as it allows for results from individual assays to be directly linked to each other using a data analysis pipeline that is integrated. A procedure to select a benchmark response (BMR), which is similar to the minimum activity threshold based on intrinsic response variation in the individual endpoints, was created. An R package to automate the process can be found at https://cran.r-project.org/web/packages/Rcurvep/index.html [last accessed 22 December 2023, v.1.2.1]. Datasets from individual assays used in the battery were reassessed with the BMC approach. BMC data of neurite outgrowth@USEPA, proliferation@USEPA, and acute neuronal firing@USEPA were collected from [23]. All other BMC data, apart from the BMC data of endpoints at IUF, were retrieved from the DNT-DIVER database (BMC model = “Curvep”) (https://doi.org/10.22427/NTP-DATA-002-00062-0001-0000-1) [last accessed 22 December 2023]. BMCs for endpoints from IUF were derived from raw data published in [21] using the same analysis method as in DNT-DIVER. The NCATS neurite outgrowth data were requested from the authors in [34]. Two chemicals (BDE-47 and BPDP) were retested in the EPA network formation assay (data accessible via EPA invitrodb 4.0). To assess DNT selectivity, the ratio between the BMC cytotoxicity and the BMC of the DNT endpoint was calculated. “Selective” was defined as when the fold change was larger than 2-fold. If the BMC of cytotoxicity could not be determined (i.e., non-cytotoxic for the tested concentration range) the BMC of cytotoxicity was set to the highest tested concentration and was considered selective despite a lower value than 2-fold since cytotoxicity was inactive.

#### 2.3.2. Human Exposure Evaluation

Human biomonitoring or exposure data were identified and collected from the literature and from data reported in the OECD IATA case study [16]. In addition to the exposure scenario reported in the OECD case study, i.e., breastfeeding, handwipes, and house dust (Supplemental Table S10 with Notes column of “OECD study”), metabolite concentrations in human urine samples were collected. The added data and corresponding references are noted as “Additional data” in the Notes column of Supplemental Table S10. To allow comparisons across various exposure scenarios, as well as to relate in vitro toxicity data to human exposure, the maximum plasma concentration (C_max_) values from different types of exposure were estimated using the httk.PBTK model, which is a generic physiologically-based toxicokinetic (PBTK) model provided by the U.S. Environmental Protection Agency (EPA)’s high-throughput toxicokinetics (httk) R package (v.2.2.2) [46]. The httk.PBTK model includes gut, artery, vein, lung, liver, kidney, and rest-of-body compartments and can simulate chemical distribution following exposure via the intravenous injection and oral dosing routes.

Before applying the httk.PBTK model for simulation, the human exposure data were firstly converted to oral doses using the available information (e.g., infant body weight, age) in the actual study, and using standard hand–mouth equations (e.g., frequency of contacts) for house dust and handwipe exposure, and standard breastmilk intake amounts. For exposure via house dust and handwipes, we assumed 18 contacts per hour over a 12 h awake period. For infant exposure via breastfeeding, we assumed the total daily breastmilk intake is 800 g/day at intervals of eight times a day for an infant of 4 kg body weight [47].

The oral dose via handwipe exposure for a child with body weight of 20.25 kg is calculated as follows [10].

Oral dose per handwipe contact (mg/kg/contact) =

$$\frac{measurement\;\left(ng/wipe\right)\times transfer\;efficiency\;\times Fraction\;of\;hand\;contacted}{10^{6}\;\left(ng\;per\;mg\right)\times 20.25\;\left(kg\;body\;weight\right)}$$

where transfer efficiency = 0.5, fraction of hand contacted = 0.1.

Exposure via house dust for a child with a body weight of 10.75 kg is calculated as follows:

Oral dose through house dust (mg/kg/contact) =

$$\frac{measurement\;\left(ng/g\right)\times total\;daily\;dust\;intake\;\left(g\right)}{10^{6}\;\left(ng\;per\;mg\right)\times 10.75\;\left(body\;weight\right)\times contacts\;per\;hour\;\times awake\;hours}$$

where total daily dust intake = 0.1 g; number of contacts per hour = 18, awake hours = 12.

Exposure through breastmilk is calculated as follows:

Daily oral dose through breastmilk (mg/kg/day) =

$$\frac{measurement\;\left(ng/g\;lipid\right)\times total\;lipid\;content\;\times total\;breastmilk\;intake}{10^{6}\;\left(ng\;per\;mg\right)\times 4\;\left(kg\;body\;weight\right)}$$

where total lipid content = 0.033 [21], total breastmilk intake = 800 g.

A period of one year of exposure (365 days) was simulated for all exposure scenarios. To run the httk.PBTK model, the “Solve_pbtk” function of the httk R package is applied to predict chemical distribution following various types of oral doses described above. To use the “Solve_pbtk” function, the user needs to provide a dosing scenario, such as dose amount, frequency, and several input chemical parameters, such as fraction of the chemical unbound to plasma, intrinsic metabolic clearance, pKa, and LogP, which were provided from the ADMET Predictor (Simulations Plus, Inc., Lancaster, CA, USA). The R script to run the “Solve_pbtk” functon can be shared upon request. The “Solve_pbtk” function is also accessible through a user-friendly interface provided by Integrated Chemical Environment (ICE, https://ice.ntp.niehs.nih.gov/) (access date 15 April 2024) [48]. In situations where measured human plasma data were available, a serum density of 1.06 kg/L was used for unit conversion. Lipid content adjustments were also applied when converting plasma measurements, given in ng/g lipid weight, to chemical concentration in total plasma [2]. The total lipid contents used were 2 g/L for cord plasma [49], 7.7 g/L for adult plasma [49], and 1.8 g/L for child plasma [10,47,50].

### 2.4. Gathering of Additional Data

As the IATA framework considers integration of all available data (Figure 1), the Integrated Chemical Environment (ICE) and the literature were explored to gather additional in vitro and small model organism data.

#### 2.4.1. Integrated Chemical Environment (ICE)

ICE (https://ice.ntp.niehs.nih.gov) (access date 15 April 2024), launched in 2017 and developed by the NTP Interagency Center for the Evaluation of Alternative Toxicological Methods (NICEATM), is an open-source web platform for accessing data and tools supporting chemical safety assessment [18,51]. ICE provides free, unrestricted access to a large collection of in vivo, in vitro, and in silico curated data, as well as computational tools to support the development, evaluation, and application of NAMs [52].

Representative OPFRs and classic BFR chemicals were inputted into the ICE search tool and queried against high-throughput (cHTS) screening data provided by the U.S. ToxCast and Tox21 initiatives (accessed 29 September 2023; ICE v4.0.1) and curated to incorporate analytical chemistry quality control information on a per-sample basis and potential technological interference flags [52]. Data from diverse biological assays/endpoints, including neurotransmission, abnormal cell growth and differentiation, angiogenesis, cellular processes, immune and inflammatory responses, endocrine disruption, xenobiotic metabolism, gene expression, and energy metabolism were extracted and evaluated. Results were sent to Curve Surfer to obtain concentration response curves. A filter to obtain only active calls was applied and activity concentration at cutoff (ACC) or lowest observed effect concentration (LOEC) was extracted for each curve and compared to the DNT BMC to determine [53] the potency of the chemicals and sensitivity of the DNT battery. The curves that had a lower ACC/LOEC than the BMCs for the DNT battery assays were manually checked for quality; curve fits that were not reliable, e.g., no data points below AC50 or excess variability, were excluded (Supplemental Table S8).

#### 2.4.2. Literature Review

Data for the OPFRs were extracted from the literature reviewed by Patisaul et al. 2021 and annotated to various neurodevelopmental processes, including endocrine disruption, apoptosis/necrosis, glial (astrocytes, oligodendrocytes, microglia) differentiation and function, neuronal differentiation and maturation (ontogeny of neurotransmitters and receptors, neurite morphology, synaptogenesis), and behavior (zebrafish and medaka) (Supplemental Table S2). Several of these processes are currently not covered in the DNT battery. Observed effects and LOECs were extracted based on individual analysis and interpretations reported in each of the studies. No bias analysis was performed. Moreover, the lowest benchmark dose (BMDL) from in vivo animal studies and estimates of exposure levels posing minimal risk to humans (MRLs) were extracted from the Agency for Toxic Substances and Disease Registry (ATSDR) when available [53].

## 3. Results

### 3.1. Most Sensitive Endpoint in Each Assay of the DNT Battery

**Figure 2:**
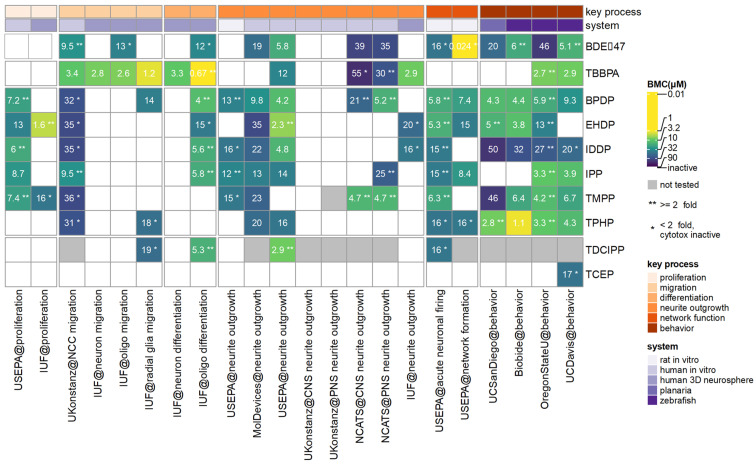
Heatmap of the various assays in the DNT battery and the ten FRs tested. Assays were organized based on their associated key neurodevelopmental process (peach shades) and cell system (purple shades). The colors and numbers in the heatmap represent BMCs (μM) of the most sensitive endpoint (MSE) measured within the assay. White color represents inactive and grey not tested. Stars indicate selective DNT effects, defined as ratio between cytotoxicity and BMC for DNT endpoint being ** >2 and * ≤2 if no cytotoxicity was observed at any concentration tested.

The heatmap shown in Figure 2 provides an overview of the most sensitive endpoint (MSE) in each assay of the DNT screening battery for the assessed FRs (Supplemental Table S5). Considering compound class, all the aromatic OPFRs were active in 11–17 of the 22 assays in the DNT battery, with 5–10 of them being selectively active (no cytotoxicity or cytotoxicity at >2-fold lower concentration). They were overall much more active than the two halogenated OPFRs (TDCIPP and TCEP) which only showed activity in one to four assays. The compound that affected most assays was BPDP (15/22), while the OPFRs that had the lowest BMC were EHDP and TPHP. However, the BFRs, BDE-47, and TBBPA, still had the lowest BMC of all compounds, which was observed in the network formation (NFA) assay for BDE-47 and oligodendrocyte differentiation and radial glia migrations for TBBPA. While the phased-out BFRs did not affect proliferation, this was a commonly affected key process for the aromatic OPFRs tested here.

Comparing the assays to one another, the oligodendrocyte differentiation, NCC migration, human US EPA NOG, acute network function and two of the zebrafish behavior assays identified eight of the FRs as active, followed by seven actives in the MolDevices NOG, six in planarian, and one zebrafish behavior assay. None of the FRs were active in the UKN CNS and PNS NOG assays and only one FR (different FRs depending on assay) was active in the IUF oligodendrocyte migration, neuronal migration, differentiation, and NOG assays. Asterisks were used to indicate selective active hits, where the ratio between the BMC of the DNT endpoint and cytotoxic BMC was >2 (≤2 if no cytotoxicity was observed). The most selective assays were oligodendrocyte differentiation, NCC migration, and OSU zebrafish behavior where 8/8, 7/8 and 7/8 of the active compounds, respectively, had selective effects. None of the seven active compounds in the MolDevices NOG were selectively active, and only 1/6, 2/6, and 2/8 were selectively active in the Biobide zebrafish behavior, planarian behavior, and human US EPA NOG, respectively.

### 3.2. Data Integration of All Endpints in the DNT Battery

**Figure 3:**
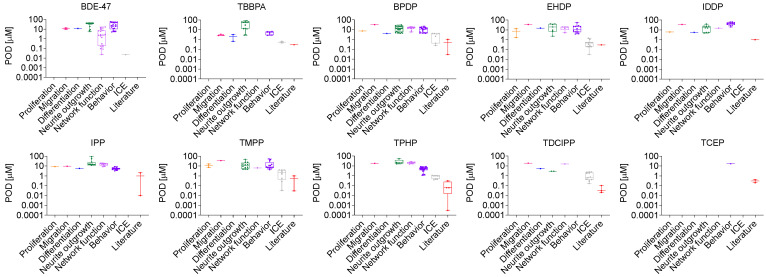
Distribution of assay endpoints. Boxplots display the distribution of all BMC hit calls (µM point of departure (POD)) for each of the endpoints in the DNT battery (organized after key neurodevelopmental process), along with ICE and literature endpoints that fell below the MSE for each chemical.

To further characterize the results of the screening in the DNT battery, the distribution of all endpoints for each assay were plotted in boxplots (Figure 3 and Supplemental Table S4). This way it became clear that the BMCs for the different assays were generally within 10-fold of one another, with only BDE-47 showing distinctly a lower BMC for several endpoints in the network formation and function assays. Most multi-endpoint assays had similar BMCs for all the endpoints within the assay. Instead, the greatest variation in endpoint sensitivity was seen between various assays measuring the same key process, such as NOG, network function, and behavior endpoints.

### 3.3. Data Integration from ICE and Literature Review

To determine how the sensitivity of the DNT battery compared to other biological endpoints, DNT MSEs were compared against ICE bioactivity data where they were available (Figure 3 and Supplemental Table S8). This included assays for abnormal cell growth and differentiation, cell stress, cell process, angiogenesis, xenobiotic metabolism, immune, energy metabolism, endocrine, neurotransmission, and gene expression. Concentration response curves through Curve Surfer in ICE were obtained for seven of the FRs, and three of the FRs (IPP, IDDP and TCEP) were marked with QC-omit (see Supplemental Table S9 for details). ACC/LOEC were extracted for 998 active calls. Of these, 54 curves (~70 curves before exclusion by manual quality control curation) had lower ACC/LOEC than the BMC in the DNT battery for all seven FRs. However, the ACC/LOEC were in general close to the BMC in the DNT battery with only TMPP and EHDP having activity concentrations 125- and 50-fold lower, respectively. The majority of the MSEs in ICE were annotated to gene expression regulation associated to xenobiotic metabolism, fatty acid signaling, and the endocrine system. In addition, immune processes appear particularly sensitive for EHDP, with angiogenesis, immune, and cell processes related to immune function, all more sensitive than the endpoints in the DNT battery.

Furthermore, the literature was evaluated for additional DNT effects including those currently not covered in the battery, as well as endocrine effects as they play a crucial role in brain development. There were multiple studies reporting effects on the endocrine system at lower or similar concentrations to the BMC of the DNT battery. Except for BDE-47, these effects were observed for all FRs where data could be extracted (no endocrine related data were found for EHDP and IDDP). Alteration in binding to the estrogen receptor (ER) and increase in 17b-estradiol (E2) and testosterone (T) production were overall the most sensitive endpoints for TMPP, TPHP, TDCIPP, and TCEP at 30- to 3700-fold lower concentrations than the MSE in the DNT battery (Figure 3 and Supplemental Table S8). Zebrafish behavior assays including additional endpoints in adult fish exposed during development, such as tap test and flee response were more sensitive than the MSE in the DNT battery for BPDP (140-fold), IPP (300-fold) and TDCIPP (100-fold). However, it is noteworthy that one of the major limitations of extracting data from the literature is the dependence upon the authors interpretations, thus data integrity and information transparency cannot be guaranteed.

**Figure 4:**
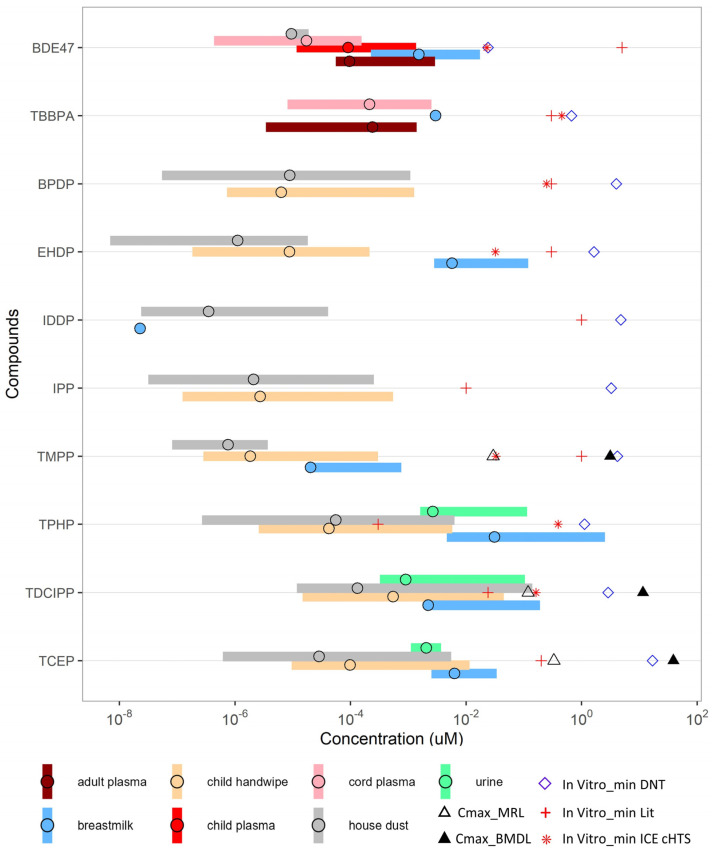
Comparison between human exposure to FRs, rodent in vivo studies, and in vitro bioactivity concentrations. The colored bars represent the ranges of human biomonitoring plasma concentration of parent compounds (dark red, red, and pink), urine concentration of metabolites (green), or C_max_ values estimated from human exposure via breastmilk (blue), child handwipe (orange), and house dust (grey) from the literature. The circles within colored bars represent the mean or median exposure values. The symbols represent BMC/BMDL/MRL (µM) from in vitro DNT assay battery (open diamond), the lowest activity concentration from in vitro assays reported in the literature (red plus), the lowest activity concentration from ICE cHTS data (red star), rat plasma concentration from the lowest benchmark dose (BMDL) (black triangle) or estimates of exposure levels posing minimal risks to humans (MRLs) (open triangle). The number values for colored bars are included in Supplemental Table S10.

The lowest benchmark dose (BMDL) from animal studies and estimates of daily exposure levels posing minimal risks to humans (MRLs) reported in the ATSDR report was extracted as a comparison to the in vitro data and human exposures [53]. Only three compounds, TMPP, TDCIPP and TCEP had sufficient studies to calculate BMDL values and only for TCEP neurological effects were the most sensitive endpoint (Supplemental Table S10). The BMDL (intermediate exposure 15–364 days) for TMPP was 3.72 mg/kg/day based on ovarian lesions in rats giving an MRL of 0.04 mg/kg/day when applying an uncertainty factor of 100 (10 for animal to human extrapolation and 10 for human variability) while chronic exposure (>364 days) lowered the BMDL to 2.12 mg/kg/day and MRL to 0.02 mg/kg/day for the same endpoint. TDCIPP gave a BMDL (intermediate exposure) of 4.69 mg/kg/day (increased kidney weight) and an MRL of 0.04 mg/kg/day while chronic exposure gave a BMDL of 1.94 mg/kg/day based on renal tubule hyperplasia and an MRL of 0.02 mg/kg/day. TCEP, the only FR where neurological effects were the most sensitive endpoint (necrosis of hippocampal neurons) gave a BMDL of 60.76 mg/kg/day (MRL 0.6 mg/kg/day) for intermediate exposure (59.86 mg/kg/day for chronic exposure). However, for chronic exposure renal tubular lesions were the most sensitive endpoint with a BMDL of 23.42 mg/kg/day and an MRL of 0.2 mg/kg/day. The lowest MRLs were plotted with the exposure data (Figure 4) to show the most protective of human health. Additional in vivo data from the literature were mainly based on mixtures or one concentration why that information was not used to extrapolate to the in vitro data [54]. Hypothalamic gene expression changes related to endocrine effects of OPFR mixtures were observed in developmental studies in mice at 1 mg/kg/day of each OPFR [55], that in a follow up study reported anxiety-like behavior at the same concentrations [56]. Moreover, 50 mg/kg/day of TPHP was shown to permeate the blood–brain barrier (BBB) and induce significant transcriptomic and metabolite changes in the brain tissue of mice [57]. Neuropathological abnormalities and decline in learning and memory was also observed in rats exposed to 100 mg/kg/day of TCEP [58].

### 3.4. Relevance to Human Exposure

To relate in vitro and in vivo toxicity data to human exposure, the literature was curated to obtain human exposure and biomonitoring data for the specific compounds, especially for infants and toddlers. The exposure sources included child handwipe, breastmilk, and house dust. The biomonitoring data included adult plasma, child plasma, cord plasma, and urine concentrations. The biomonitoring plasma and urine data were compared to in vitro activity concentration data directly and estimated rat in vivo plasma concentration after serum density adjustment (Figure 4) using the EPA’s httk.PBPK model.

The human exposure from different sources varied largely, covering a few orders of magnitude difference. Of different exposure sources, the exposures through breastmilk (blue bar, Figure 4) were the highest, followed by handwipe (orange bar) and house dust (grey bar) exposures, which were more similar. Within the same type of human exposures, the variation ranges 1–3 orders of magnitude across FRs. The C_max_ values estimated from house dust and handwipe exposures are >10-fold lower than the lowest in vitro activity concentrations for all the FRs, except TPHP and TDCIPP. For TDCIPP, the C_max_ value estimated from the highest house dust and handwipe exposure approximates the lowest ACC values or lowest observed effect concentrations from ICE cHTS data and overlap with the LOEC from the literature and the MRL from in vivo rat studies. Moreover, BMCs from the DNT battery showed similar values to estimated rat plasma concentrations from in vivo BMDL data for TMPP, TDCIPP, and TCEP. Overlaps between C_max_ values estimated from breastmilk exposure and the lowest in vitro activity concentrations were also observed for three FRs (i.e., EHDP, TPHP, and TDCIPP). In addition, the plasma C_max_ estimated from breastmilk exposure approximate the lowest in vitro activity concentration for BDE-47 and is about 10-fold lower for TCEP (Figure 4). These data suggest that some human exposure is high enough to lead to an internal (e.g., plasma) concentration similar to the concentration that is bioactive in vitro.

## 4. Discussion

Systematic testing for DNT is not routinely required by regulatory agencies but becomes mandatory when obvious neurotoxicity is observed during organ system toxicity testing [59]. Therefore, only a small number of DNT toxicants are known and fewer are regulated. Test guidelines from the U.S. EPA and OECD have been developed to function as a framework for regulatory screening and further test for the DNT potential of chemicals. However, due to high costs, lengthy time requirements, the high number of animals used, and methodological and/or scientific uncertainties, testing thousands of potential DNT chemicals is not feasible [12]. These issues pertaining to DNT testing and the increased need to carry out the hazard assessment of potential DNT chemicals has led to calls for the development and use of in vitro methods. A DNT—in vitro battery (IVB) was thus established through a consensus of scientific stakeholders from regulatory agencies, academia, and industry and is described in the OECD guidance document [15]. To exemplify how the DNT–IVB data can be used in regulatory applications, several IATA case studies were developed for various purposes, including hazard characterization and identification [60], prioritization [16], or waiving of an in vivo guideline study [61].

In this IATA case study, the aim was to prioritize aromatic OPFRs for further DNT in vivo testing. Two BFRs and two halogenated OPFRs were included to compare their relative toxicity to determine if the OPFRs are better substitutes.

### 4.1. Comparison of Potency across Phased-Out BFRs, Halogenated FRs, and OPFRs

#### 4.1.1. Brominated FRs

It is well documented that exposure to BFRs and their metabolites can induce neurodevelopmental effects such as lower IQ, impaired cognition, and behavior issues [62,63,64]. Within the DNT battery, BDE-47 was the most potent of all FRs but only due to the NFA being about 200 times more sensitive than the other DNT battery assays that otherwise had similar BMCs for all FRs. There is overlap among some of assays in the DNT battery and ICE and the NFA assay was also the MSE reported in ICE. There were limited data extracted from the literature for BDE-47, as the primary focus for that search was the OPFRs. TBBPA was the second most potent of all FRs. The MSE was oligodendrocyte differentiation, followed by radial glia migration. The MSEs in ICE for TBBPA were gene expression regulation of peroxisome proliferator-activated receptor gamma (PPARγ) with similar activity concentration as the oligodendrocyte differentiation. PPARγ activation was identified as one of the MSEs in the literature at similar concentrations as in the ICE gene expression and was a hit for seven of the flame retardants assessed here—BDE-47, TBBPA, TPHP, EHDP, TMPP, BPDP, and TDCIPP. Though PPARγ is mainly recognized to be involved in fatty acid signaling, it has also been shown to be essential in regulating early brain development (reviewed in [65]) and neurogenesis in the adult brain [66]. The receptor has shown to play a role in the differentiation and function of oligodendrocytes [67] and has been identified as relevant for endocrine disruption-induced DNT using in silico predictions [68]. Using TBBPA as a stressor, Klose et al. [69] developed a putative AOP with an unknown molecular initiating event (MIE) leading to altered cholesterol metabolism, followed by reduced number of oligodendrocytes. Studies have shown that PPARγ regulates cholesterol influx, efflux, and metabolism [70] and could be the missing MIE. In summary, the DNT battery alone was able to identify the two BFRs as developmental neurotoxicants, while integration of additional assays from ICE and the literature supported identification of potential mechanisms.

#### 4.1.2. Halogenated FRs

The two halogenated OPFRs were less active than the two other classes. However, there is a considerable uncertainty in the case of TDCIPP, as it was only tested in 10 of the 22 assays. There were no ACCs/LOECs derived for TCEP in ICE due to the compound being labeled QC-omit (low purity). Manual evaluation of the curves still indicated similar activity concentrations as the DNT battery. TDCIPP was active in several of the assays in ICE with assays for gene expression regulation of the Pregnane-X receptor (PXR) being most sensitive at 10–20-fold lower concentrations than the DNT battery. PXR is a key regulator in the xenobiotic response pathway as it regulates the expression of various drug metabolizing enzymes, such as P450s and glutathione S-transferases [71]. However, activation of xenobiotic nuclear receptors, including PXR, has also been identified as an MIE in AOP8 (https://aopwiki.org/aops/8) (access date 15 April 2024) leading to thyroid hormone disruption that consequently can result in altered neurodevelopment. The literature review supports these findings as the MSEs were related to alteration in estradiol and thyroid production at 30 (TCEP) to 140 (TDCIPP) times lower concentrations than the DNT battery. TCEP also induced a decrease in expression of oligodendrocyte markers in zebrafish embryos at similar concentrations [72]. Oligodendrocyte differentiation and maturation is dependent on thyroid signaling that further supports these findings [73,74]. However, oligodendrocyte differentiation was not affected by TCEP in the DNT battery, indicating that an intact organism such as zebrafish could provide additional information not necessarily picked up by in vitro assays such as secondary DNT effects due to other organ toxicity. On the other hand, TDCIPP did affect oligodendrocyte differentiation, implying that these two compounds have different targets. TDCIPP was not tested in any of the small organism behavior assays, but the literature review identified the zebrafish locomotor assay as one of the most sensitive at 50-fold lower concentrations than the MSE in the DNT battery [75], supporting again the supplementing of the OECD DNT–IVB with such assays. In summary, halogenated OPFRs evaluated here seem less of a concern for DNT effects based on the DNT battery. However, integration with additional data indicate endocrine effects that could lead to DNT, a mechanism with limited coverage in the current battery.

#### 4.1.3. Aromatic FRs

As a class, the aromatic OPFRs were at least as active as the BFRs in the DNT battery. Active in the greatest number of assays in the DNT battery was BPDP, with similar BMCs [76] (4–32 μM) and at least one active assay in each neurodevelopmental key process (Figure 2). The MSE in ICE for BPDP was gene expression regulation of the PXR which was 15 times more sensitive than the MSE in the DNT battery. As discussed previously, PXR is a potential MIE in AOP8 (https://aopwiki.org/aops/8) (access date 15 April 2024) that can lead to altered neurodevelopment through thyroid hormone disruption. Limited amounts of data were identified in the literature for BPDP, with the MSE observed in a study using zebrafish behavior assays [76]. In this study, effects were observed at a lower concentration than the MSEs in the DNT battery, including the zebrafish behavior assay. However, the MSEs were observed in different endpoints such as the novel tank test, tap test, shoaling and predator avoidance, which indicate long-term impairment of anxiety-related behavior in the zebrafish. The significant distinction lies in the assessment duration. Glazer et al. employed a detailed and time-intensive approach, evaluating lifetime effects on the nervous system in adulthood following developmental exposure. Conversely, the DNT screening battery is designed for rapid and efficient screening during the larval stage, emphasizing rapid identification and prioritization of DNT compounds. Moreover, the experimental designs in these studies were different, with the most prominent being the exposure scheme as the zebrafish assays in the DNT battery only dose the animals one time while the Glazer et al. study renewed the exposure solution every 24 h leading to cumulative exposures.

The aromatic OPFR TMPP had the second greatest number of active assays in the DNT battery. In ICE, the MSE assay (125-fold more sensitive vs. DNT battery) was binding to the peripheral benzodiazepine receptor (PBR), also known as 18 kDa translocator protein (TSPO). TSPO is involved in the regulation of cholesterol transportation and is the rate-determining step in steroid biosynthesis (reviewed in [77]), a critical component for brain function (reviewed in [78]). The literature review identified that TMPP exposure induced alteration in binding of 17b-estradiol (E2) to the estrogen receptor and changes in E2 and testosterone production [79]. It has been shown that alteration in estradiol signaling in primary cultures of astrocytes can also lead to modulation of TSPO activation [80], which is why this mechanism might be important to explore. Currently, only the rat primary cell models in the DNT battery have astrocytes present but their function has not been characterized, indicating a gap that could potentially be important to address. The neurosphere assay from IUF includes radial glial cells that can later differentiate into astrocytes, but their function is very different from astrocytes as they mainly guide neurons to their correct position during migration, while astrocytes support various processes such as neurogenesis, synaptogenesis, blood–brain barrier permeability, and maintaining extracellular homeostasis (reviewed in [81,82]).

EHDP, IDDP, and IPP were all active in half of the assays in the DNT battery. EHDP induced a slightly lower BMC than the others, having the second lowest MSE among the aromatic OPFRs. Within ICE, the MSE was enzymatic activity of matrix metalloproteinase (MMP)-9 with activity concentrations 50-fold lower than the DNT battery. MMP-9 is most associated with its role in degrading extracellular matrix components in cancer (reviewed in [83]). However, in the brain, MMP-9 controls the shape of dendritic spines and function and contributes to synaptic plasticity, subsequently playing a critical role in learning and memory (reviewed in [84]). Moreover, MMP-9 has been shown to be involved in neuroinflammation and various neurological disorders (reviewed in [85]). EHDP was active in several other assays in ICE annotated to inflammatory response at 10-fold lower concentrations than the DNT battery, supporting the need to characterize and/or add cells with immune response. In the literature review, the MSEs were the zebrafish behavior assays for IPP and EHDP [76] at 300- and ten-times lower concentrations than the zebrafish locomotor assay in the DNT battery, again likely due to the cumulative exposure scheme and evaluation during adulthood. IDDP seems to be the least potent of all the aromatic OPFRs, based on the DNT battery and the literature review. Neither IPP nor IDDP had ACCs/LOECs calculated in ICE as their purity was considered low (Supplementary Table S10). In summary, the aromatic OPFRs had similar activity to the BFRs in most of the assays in the DNT battery (NFA being an exception), indicating that the OPFRs may not be a safer replacement for certain phased-out BFRs. The DNT battery was sufficient to indicate a concern for DNT. However, the integration of data from ICE and the literature suggests that the point of departure for this class of compounds may be lower if endpoints for endocrine effects were included, likely due to endocrine effects being upstream of the key processes measured in the DNT battery.

### 4.2. Comparison of In Vitro and Small Model Organisms Data to Human Exposures and In Vivo Studies

To contextualize findings from the in vitro and small model organism assays for risk assessment, we compared the activity concentrations in the DNT battery to the biomonitoring plasma concentration, estimated plasma C_max_ from various human exposure scenarios or the BMDL, and estimated MRL from rat in vivo studies. We observed similar plasma C_max_ values estimated from breastmilk intake to the lowest in vitro activity concentration for several FRs—notably BDE-47, EHDP, TPHP, and TDCIPP (Figure 4). This observation suggests that human exposure, might pose a risk for neurodevelopment and endocrine-related effects—the MSEs for these compounds. Similarly, the currently set MRL for TDCIPP overlap with estimated human exposures and support a potential concern, especially as the MRLs were based on studies in adult animals. It should be noted that, even though the risk appears to be lower for the remaining compounds, there are limited exposure data for most of the aromatic OPFRs.

There are some factors that may contribute to the uncertainty when estimating C_max_ from human exposure. When experimental values are not available, predicted values for pharmacokinetic parameters, e.g., fu and intrinsic clearance, and default model assumptions regarding absorption and bioavailability were used, which may not accurately represent the pharmacokinetics of each chemical. Inter-individual variability in human physiology and how their bodies absorb, distribute, metabolize, and excrete chemicals also exists, leading to variations in plasma C_max_ values among individuals. Limited information about the bioavailability of chemicals in the in vitro assay systems (e.g., how these chemicals interact with plasticware and cell media components) adds another layer of uncertainty to predictions. In addition, the process of converting exposure data from various sources to daily oral exposure (mg/kg/day) involves specific assumptions. Application of different assumptions would lead to different predictions on C_max_. A better understanding on exposure sources and refining the conversion processes might improve estimation accuracy of C_max_.

### 4.3. Chemical Considerations

Herein, we evaluated only the parent compound; if the major metabolites were to be included, the expected total exposure would be higher. Since major metabolites (e.g., DPHP, ip-DPHP) are not specific to one parent compound, it makes it challenging to associate one specific compound with a unique metabolite. With the phasing out of the BDEs and the proposal to ban halogenated flame retardants, the use of aromatic flame retardants is expected to be on the continual rise [86]. Furthermore, it has been shown that children have a higher burden to flame retardants compared to mothers, thereby raising concerns for DNT [87]. The current approach has not accounted for accumulating effects from multiple sources of exposure, exposure to sensitive populations, and exposure to mixtures of OPFRs, which is typically how they exist in the environment as several flame retardants are present in a commercial mixture. This is important because, while we do not know the potential health effects of these OPFRs, they have patterns of in vitro activity at comparable concentrations to those of the phased-out or well-studied flame retardants (e.g., BDE-47 and TBBPA). Furthermore, each of these flame retardants could be acting on the same process at once, so the bioactive concentration could actually be the accumulation of their individual effects.

The concerns of mixtures also bring up questions of metabolism, as many of these compounds are readily degraded into their base compound [88]. For instance, TDCIPP can undergo metabolism via hydrolysis and oxidative dehalogenation. These metabolites may pose greater toxicity than their parent compound. While metabolism has been assessed for some of the OPFRs, there is overall a very limited understanding of metabolism of these compounds. In order to gain a general appreciation for metabolic and toxicity potential, a recent report has provided predictions for metabolites and their metabolite priority scores for all flame retardants in, or formerly in commerce [89]. The authors similarly emphasized the concern of accumulating exposures of metabolites from various parent compounds.

### 4.4. Prioritization for Further Testing

The goal of this case study was to prioritize aromatic OPFRs for further DNT in vivo evaluation based on the in vitro and small model organism data. Looking at the obtained data, there is no compound that clearly stands out and would therefore be prioritized. There are several ways compounds could be prioritized. One option is to consider the MSE (lowest BMC in any assay), which would prioritize TPHP and EHDP. The BFRs were still ranked higher than all OPFRs based on the MSEs, with the BMC for BDE-47 being almost 30-fold lower than all the other FRs, however, this is only based on one assay, the NFA.

Other options would be to look at the most hits or most selective hits (activity in DNT endpoint without cytotoxicity), which would have prioritized BPDP. However, selective vs. non-selective activity may need to be interpreted differently for the various endpoints as the experimental design varies, e.g., some assays assess cytotoxicity at the same time as the DNT endpoint, while other assays only assess the cytotoxicity in the end of the experiment with a combined measurement over several days for the DNT endpoint. A change in time point for cytotoxicity assessment in the experimental design could potentially change the selectivity of the endpoint and such interpretation should therefore be conducted with caution. In the prioritization approaches described above, each assay has equal weight. However, it can be considered to give assays closer to the adverse outcome, e.g., behavior and functional assays, a higher weight. In this case, BPDP, IDDP, TMPP, and TPHP were active in all four behavior assays (zebrafish and planarian), and BPDP and TMPP were selectively active in three of the behavior assays and would therefore be prioritized. If the functional in vitro assays had higher weight, IPP would be prioritized as it was selective active in both neuronal network function assays (acute and developmental). Instead, if looking from the risk perspective and including the exposure data, TPHP would be prioritized as it had the highest predicted exposure value which overlapped with in vitro activity concentrations. As the aromatic OPFR in general had similar activity in the DNT battery, another option would be to look at the chemical structure and select dissimilar structures in cases that influence the mode of action. Taking the majority of these approaches into consideration would rank TPHP the highest on the prioritization list. TPHP is the base compound in the synthesis of the other aromatic OPFRs and would therefore be another valid reason to evaluate this compound further.

### 4.5. Uncertatinites and Future Directions

It is important to consider the uncertainties when evaluating the data in the IATA, especially if the information will be applied to make regulatory decisions. Some uncertainties identified in this IATA case study include different models resulting in different outcomes, e.g., zebrafish behavior assays and neurite outgrowth assays. It becomes important to understand protocol parameters and models that may impact different outcomes and enhance harmonization among similar assays. Within the OECD DNT expert group, there are ongoing efforts to harmonize zebrafish behavior assays. Various cell models that are evaluating the same key event should be better characterized, e.g. for coverage of biological pathways and windows of development to enhance clarification of the differences.

Integration of various data sources using different data analysis pipelines is another uncertainty, especially when incorporating literature studies as the raw data are rarely available. The requirement of making such data available and following the FAIR principles (findable, accessible, interoperable, and reusable) will allow harmonized analysis and improve interpretation of integrated data.

Uncertainties using in vitro models, however unspecific to DNT, is the low or unknown metabolic activity compared to in vivo studies. The selection of compounds, therefore, needs to be carefully evaluated and consideration of test metabolites may be necessary.

The PBK models explored here have uncertainties including utilizing assumptions such as absorption, clearance, and bioavailability, lack of consideration of inter-individual variability, and lack of BBB. There is ongoing work towards enhancing the PBPK models for these parameters that will provide better predictions and translations for IVIVE.

Furthermore, there are biological uncertainties using the DNT battery as some key processes of neurodevelopment have limited coverage. Here we identified endocrine disruption as an important mechanism that should be considered in a DNT battery. Incorporating additional assays could decrease the uncertainties and increase confidence in the IATA for DNT.

Finally, there are translational uncertainties as species difference is well recognized in DNT and limited data on human DNT effects of environmental compounds are available. Hence, cross-disciplinary collaboration among toxicologists, NAMs developers, epidemiologists, and clinicians are recommended to enhance and verify human translation.

## 5. Conclusions

In conclusion, this paper addresses concerns regarding the impact of environmental chemicals on the increase in neurodevelopmental disorders and advocates for more stringent regulations. The OECD DNT–IVB is proposed as a valuable initial screening tool for prioritizing chemicals lacking data, with a focus on OPFRs in this IATA case study. While the DNT battery highlights similarities between aromatic OPFRs and phased-out flame retardants, the limited mechanistic information prompts the need for an integrated approach using in vitro assays, literature, and IATA to identify gaps in neurodevelopmental process coverage.

Emphasizing the necessity for additional endpoints, such as endocrine disruption and the inclusion of astrocytes and microglia cell populations, this study suggests that combining the OECD DNT–IVB with NAMs could improve confidence in DNT assessment. Validation efforts for thyroid in vitro assays [90] and ongoing projects like Horizon 2020 ENDpoiNTs are recognized for their contribution to developing assays for endocrine disruption in DNT [68].

Observations in rodents and humans exposed to OPFRs also indicate DNT effects [56,58,88,91,92,93,94,95] and endocrine disruption [55,56,96].

The proposal to enhance the OECD DNT–IVB with behavior assays in zebrafish for identifying compounds with diverse modes of action is acknowledged, recognizing the sustainability challenge and potential false positives in higher-throughput screening.

There is ongoing effort to develop additional assays that will cover some of the identified gaps, e.g., glial function [97]. However, to even run all assays in the current OECD DNT–IVB is not feasible for higher-throughput screening strategies, which is why a refinement of the number of assays and/or a tired approach need to be developed. To select the most relevant assays for a refined DNT battery current cell models need to be further characterized and ongoing efforts to screen additional compounds and explore a broader chemical space are crucial.

Overall, this IATA study indicates that the OPFRs may not effectively replace certain phased-out BFRs, and in some instances, they might exhibit higher potency, particularly as we accumulate additional exposure data.

## Figures and Tables

**Table 1:** Chemicals assessed in the IATA case study.

CAS	Chemical Name	Chemical ID
5436-43-1	2,2′4,4′-Tetrabromodiphenyl ether	BDE-47 ^1^
79-94-7	3,3′,5,5′-Tetrabromobisphenol A	TBBPA
115-86-6	Triphenyl phosphate	TPHP
68937-41-7	Phenol, isopropylated, phosphate (3:1)	IPP ^2,3^
1241-94-7	2-Ethylhexyl diphenyl phosphate	EHDP ^2^
1330-78-5	Tricresyl phosphate	TMPP ^2^
29761-21-5	Isodecyl diphenyl phosphate	IDDP ^3^
56803-37-3	tert-Butylphenyl diphenyl phosphate	BPDP ^2^
13674-87-8	Tris(1,3-dichloro-2-propyl) phosphate	TDCIPP
115-96-8	Tris(2-chloroethyl) phosphate	TCEP ^3^

^1^ Phased out flame retardant. ^2^ These are not single chemicals but instead, an isomeric mixture obtained as a result of the manufacturing process. ^3^ QC-OMIT grade F in the Integrated Chemical Environment (ICE) (Caution_purity).

## Data Availability

The data presented in this study are available on request from the corresponding author.

## Supplementary Materials

The following supporting information can be downloaded at: https://www.mdpi.com/article/10.3390/toxics12060437/s1, Table S1: Identity and purity of Chemicals; Table S2: Extracted exposure data and most sensitive endpoint (MSE) ranking (concentration in uM); Table S3: All endpoints of cytotoxicity data with their different assays and data source; Table S4: All BMCs from endpoint; Table S5: most sensitive BMC by function and institute; Table S6: MSE; Table S7: MSSE2; Table S8: ICE Active calls; Table S9: ICE QC-Omit; Table S10: Biomonitoring Data. References [98,99,100,101,102,103,104,105,106,107,108,109,110,111,112,113,114,115,116,117,118] are cited in the Supplementary Materials.
